# Biomass use and its health effects among the vulnerable and marginalized refugee families in the Gaza Strip

**DOI:** 10.3389/fpubh.2023.1129985

**Published:** 2023-04-06

**Authors:** Maher Elbayoumi, Ahmed Hassan Albelbeisi

**Affiliations:** ^1^Energy and Sustainable Environment Center, School of Engineering, Israa University, Gaza Strip, Palestine; ^2^Medical Services Directorate, Gaza Strip, Palestine; ^3^College of Health Professions, Israa University, Gaza Strip, Palestine

**Keywords:** solid fuel, indoor air pollution, health, choice determinants, environmental sustainability

## Abstract

**Introduction:**

Biomass fuel remains the most common type of fuel used in many developing countries, leading to indoor air pollution and serious health impacts.

**Objective:**

The objective of this study was to compile evidence on the impact household fuel combustion has on child and adult health, with an emphasis on solid fuel use in Gaza.

**Methods:**

In this cross-sectional study, 110 structured self-administered questionnaires were distributed in April 2019 among families living in the Al-Maghazi refugee camp.

**Results:**

Participants reported that the main fuel used were wood, coal, cardboard, and a mix of wood, cardboard, and plastic, which were used for cooking, heating, baking, boiling water, and lighting. The most common symptoms were nasal irritation (71.8%), followed by headache (66.4%) and dizziness (65.4%). The results of logistic regression showed that the participants who used wood fuel had a higher chance of feeling eye irritation than those who used a mix of wood, cardboard, and dried grass (OR = 1.316; 95% CI = 1.54–8.99). The participants who opened windows during the burning process of biomass fuel were five times more likely to develop pneumonia than those who closed windows (OR = 5.53; 95%CI = 11.60–19.0).

**Conclusion:**

there is an urgent need for community awareness campaigns designed to inform people about the risks of exposure to biomass fuel smoke and how to better implement household ventilation.

## Introduction

Globally, over 3 billion people depend on biomass derived from many natural sources, plants, and animals, which are intentionally burned for several purposes, such as cooking, lighting, and home heating in developing countries (1, 2). Traditional biomass represents 13% of the world’s primary energy use and accounts for more than one-half of domestic energy in many developing countries and as much as 95% in some lower-income ones (3, 4). According to the sustainable development goal (SDG7), globally, 2.3 billion people will still be using biomass in 2030 if the current pattern continues (5).

The World Health Organization (WHO) identified indoor air pollution (IAP) induced by biomass use as one of the top 10 risks for the global burden of diseases. Household air pollution caused by the inefficient use of solid fuels was estimated to be responsible for 3.8 million premature deaths globally (6). The IAP produced by using solid fuel leads to 27, 18, 27, 20, and 8% of deaths from pneumonia, stroke, ischaemic heart disease, chronic obstructive pulmonary disease (COPD), and lung cancer, respectively (7). The adverse health effects associated with biomass fuel use include the following: low birth weight (8), cataracts (9), cardiovascular disease (CVD) (10), asthma (11), tuberculosis (12), chronic obstructive pulmonary disease (COPD) (13), and lower respiratory tract infections (14).

Household use of biomass for cooking and heating is the most widespread source of indoor air pollution among the world’s poorest and most vulnerable groups (15, 16). The Palestinian society suffers from numerous types of poverty and deprivation. The Gaza Strip, with 360 square kilometers and a population of 2.2 million, has been subjected to several political conflicts, resulting in catastrophic economic, health, psychological, social, and environmental aspects. According to a study on poverty in the Palestinian territories for the Central Bureau of Statistics (PCBS) on monthly consumption patterns in 2020, 38.8% of Palestinians in the Gaza Strip suffer from extreme poverty. Furthermore, in 2017, an Islamic Relief- Palestine (IRPAL) study indicated that the majority of the monthly income among Gaza Strip families does not exceed 160$ US dollars and that 69% of the families’ heads are jobless. In addition, 24% of the families lack the minimum standards of public health, personal hygiene, privacy, human dignity, and adequate health facilities (17).

PCBS’ Household Energy Survey revealed that the percentage of the population in the Gaza Strip relying on solid fuels for cooking and heating was 29.7 and 29.2%, respectively (18). However, this baseline could have increased from 2015 either due to the shortage in energy-efficient fuels, such as liquefied petroleum gas (LPG), and electricity, or due to the increase of alternative fuel prices. In addition to poverty, population growth increases the use of biomass fuels in the Gaza strip among vulnerable and marginalized families. The purposes of this study were: (i) to assess the types of fuels used by low and middle-income refugee families (LMIFs) in the Gaza Strip to meet household energy needs; and (ii) to compile evidence on the impact household fuel combustion has on child and adult health, with an emphasis on solid fuel use in Gaza. Therefore, this study, as far as we are aware, is considered the first attempt to characterize the dimensions of the current situation regarding the impact of using solid fuel on indoor air quality (IAQ) and health symptoms in the study area. The results obtained are a useful contribution to formulating public health policy and implementing prevention programs aimed at providing healthier environments and good quality of life.

## Materials and methods

### Study design and setting

**Figure 1:**
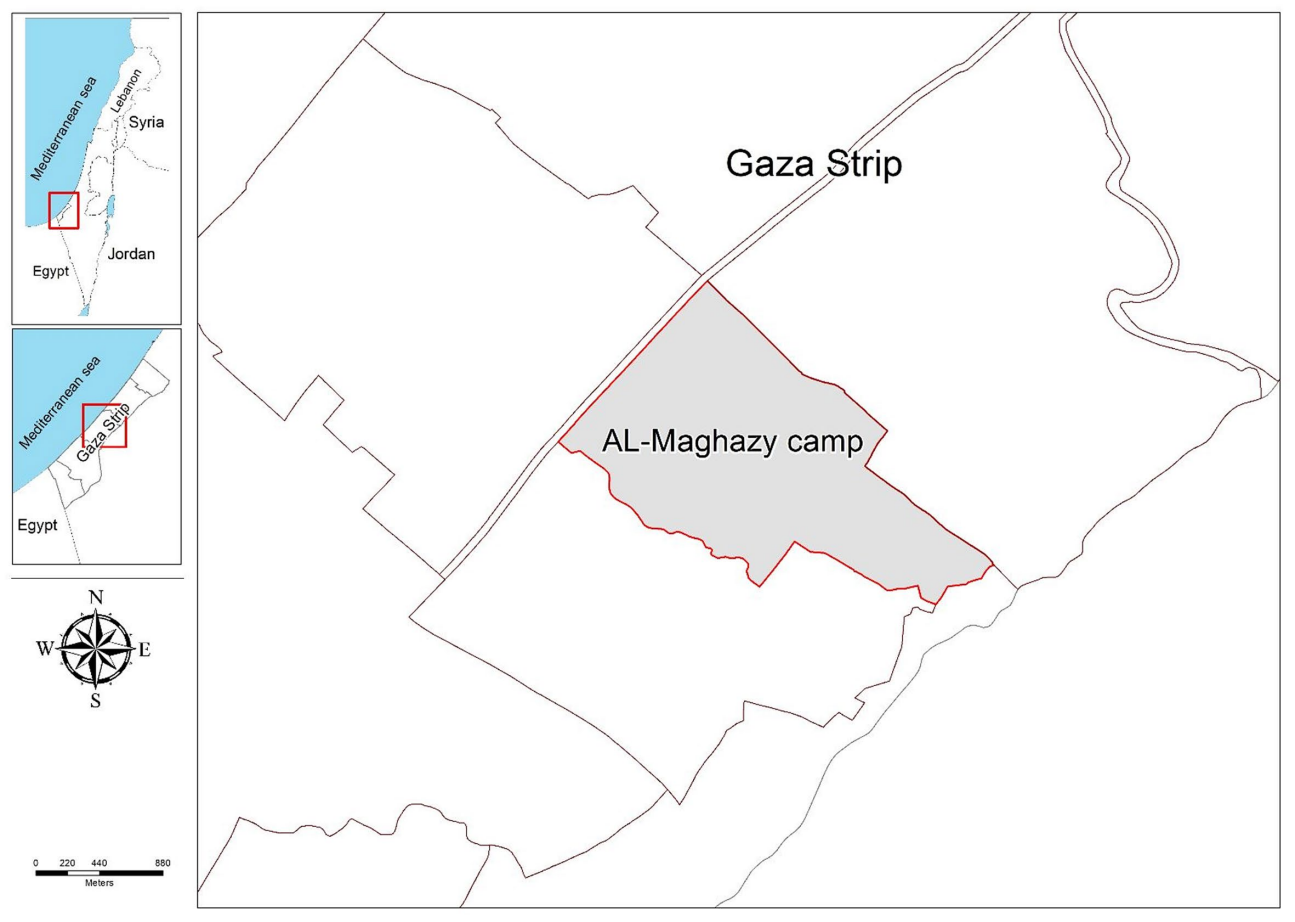
Map of the Al-Maghazi Camp.

In this cross-sectional study, a structured self-administered questionnaire was distributed in April 2019 among families living in the Al-Maghazi refugee camp. The camp, which was established in 1949, is located in the center of the Gaza Strip in the Occupied Palestinian territories, as shown in Figure 1. The camp is characterized by very narrow roads and high population density, with more than 20,105 refugees housed in an area of no more than 0.6 square kilometers (19). Thus, overcrowding, lack of living space, lack of recreational and social space, and substandard conditions are the main features of the Al-Maghazi camp. According to the United Nations Relief and Works Agency (UNRWA), the top major problems faced by refugees in the AL-Maghazi camp are poverty, unemployment, housing shortage, and lack of adequate electricity supply (20).

### Study population and sampling strategy

The study population was adult Palestinian refugees living in the Al-Maghazi camp during the data collection period, who used biomass fuel and were willing to participate in the study. All refugees who were willing to participate during the data collection period (1 month) were included.

### Data collection

Since refugee houses are similar in design and construction; the first home was chosen as the closest to the community health center and, by asking neighbors, a snowball sampling strategy was then used to select the other homes that used biomass fuel. Then, neighbors living in a residence next door who met the inclusion criteria were interviewed. If the selected family refused to participate or could not be found, neighbors living in the next residence who met the inclusion criteria were recruited.

Contact was made with families through fieldworkers and the project was explained to them. They were invited to participate in the study, received detailed information, and were asked to sign an informed consent form. Of the 153 households complying with the inclusion criteria, 110 (72%) agreed to participate. The study was carried out in April 2019.

### Study instrument

The questionnaire used was a modified questionnaire based on the adult questionnaire of the European Community Respiratory Health Survey II (ECRHS II) that has been widely used in different studies (21). The questionnaire consists of three parts: the first part of the questionnaire is meant to gather the subject’s personal and socio-demographic data; the second part of the questionnaire elicits information on descriptions and determinants of the use of household fuels; and the third part of the questionnaire is meant to gather information regarding the risk factors of health-related complaints and symptoms in the residential environment in the past 3 months.

### Validation of the questionnaire

The questionnaire was translated into Arabic by two independent translators and the two versions were merged into one preliminary version. Then, the Arabic version was back translated into English and both the old and new versions were compared. Face and content validity were checked for the final Arabic draft questionnaire. To validate the questionnaire after the translation process, it was presented to a jury of associate professors from the Islamic University of Gaza who are specialists in environmental engineering, public health, and school health education. The items of the questionnaire were modified according to their recommendations. Then, questionnaires were distributed among 30 adult residents for the piloting study to measure the reliability of the questionnaire. To confirm the reliability of the questionnaire Cronbach’s alpha technique (0.759) and split-half technique (0.791) were used (22).

### Data analysis

Data were analyzed using the software IBM-SPSS (Statistical Package for Social Sciences), version 22. Descriptive statistics summarized the Characteristics of respondents. Percentages and frequencies were used to summarize categorical variables. In addition, Chi-square, Fisher’s exact test, and logistic regression were used for data analysis. Results are presented as ORs with 95% confidence intervals (CIs). Logistic regressions were applied to test the association after adjusting for covariates. The core covariates considered in the models were gender, number of family members living in the same house, education level, and number of smokers at home. All analyses were conducted using SPSS Version 24 (IBM SPSS Statistics, Chicago, IL, United States).

### Ethics approval and consent to participate

This study was approved by the Energy and Sustainable Environment Centre, Israa University. Furthermore, informed consent was collected from each participant before data collection.

## Results

### Characteristics of the study respondents

The characteristics of respondents are summarized in Table 1. A total of 110 household refugees completed the survey questionnaire. The majority of the respondents were men (79.1% men vs. 20.9% women). In addition, the participants’ families were considered as being large families, 80.0% of them, if they had more than six persons. Around 70% of the respondents had a total monthly income of less than 1,500 NIS (400 $). In terms of husband and wife education, nearly a third of them had a university degree (33.6, and 35.5%, respectively). In terms of smoking habits, approximately 48.2% of the households had at least one member who smoked tobacco.

**Table 1:** Characteristics of the study participants.

Variables	*N* (%)
**Sex**	
Male	87 (79.1)
Female	23 (20.9)
**Family members**	
5≤ persons	22 (20.0)
6≥ persons	88 (80.0)
**Income/NIS**	
Less than 1,500	76 (69.1)
Between 1,500–2,500	23 (20.9)
More than 2,500	11 (10.0)
**Have infant inside the home**	
Yes	38(34.5)
No	72(65.5)
**Husband education level**	
Primary	42(38.2)
Secondary	31(28.2)
University	37(33.6)
**Wife education level**	
Primary	27(24.5)
Secondary	44(40.0)
University	39(35.5)
**The number of smokers at home**	
No one smokes	57(51.8)
One	31(28.2)
Two or more	22(20.0)

NIS, New Israeli Shekel.

### Determinants for using household fuels

#### Types of solid fuel and frequency of use

A wide variety of solid fuels are used in the study area for cooking and heating. Table 2 shows how different types of fuels are used in homes. Participants reported that the main fuels used were wood, coal, cardboard, and a mix of wood, cardboard, dried grass, and plastic, 30.9, 4.5, 5.5, and 59.1%, respectively. These fuels are often collected from the roadside and the local environment due to the availability of agricultural lands that surround the Al-Maghazi camp.

**Table 2:** The use of biomass fuel.

Items	Frequency (%)
**Type of biomass fuel used**	
Wood	34 (30.9)
Coal	5.0 (04.5)
Cardboard	6.0 (05.5)
Combination of wood, cardboard, dried grass, and plastic	65 (59.1)
**Frequency of using biomass fuel**	
Once a day	30 (27.3)
More than once a day	38 (34.5)
Once a week	20 (18.2)
More than once a week	22 (20.0)
**Duration of biomass fuel use/day**	
One hour or less	26 (23.6)
Two hours	49 (44.5)
Three hours or more	35 (31.8)
**Location where biomass fuel is burned**	
Inside the home	31 (28.2)
Outside the home	79 (71.8)
**Purpose for using biomass fuel**	
Cooking	21 (19.1)
Making bread	16 (14.5)
Boiling water	4.0 (03.6)
Heating	17 (15.5)
Lighting	2.0 (01.8)
All (cooking, making bread, boiling water, heating, and lighting)	50 (45.5)
**Windows during biomass burning process**	
Open	97 (88.2)
Closed	13 (11.8)

The effects of exposure to indoor air pollution depended on the duration of the use of biomass energy for cooking and other purposes per day. Table 2 shows that more than two-thirds (76.3%) of the study participants used biomass fuels for more than 2 h each day and 23.6% used them for an hour or less each day. Furthermore, 62.0% of participants reported that they used biomass fuel once or more daily. Thus, the percentage of biomass energy emissions that reaches people’s breathing zones is much higher than for outdoor sources.

### Location and estimated biomass fuel weight used each time

Exposure effectiveness of indoor air pollution depends on several factors such as dispersion of pollutants, ventilation, and duration of exposure. Of the study participants, 35.4% of them burned biomass inside the building, 41% burned biomass outside the building, and 23.6% burned biomass on the roof of the building. A majority, 88.2% of the study population, opened windows during the biomass burning process. During the burning process, the combustion of unprocessed solid fuel stoves is emitted outside, which increases pollutant concentration that significantly affects the local “neighborhood” pollution levels (23). The ventilation rate may influence indoor concentrations since infiltration is driven by pressure gradients that are affected by both wind direction and speed.

### The purpose and reasons for biomass fuel use

Table 2 shows that the main use of energy in homes among the study population was for cooking only (19%), followed by heating (15.5%), baking (14.5%), boiling water (3.6%), and lighting (1.8%). Furthermore, 45.5% of the respondents revealed that they used biomass for all the abovementioned purposes.

**Figure 2:**
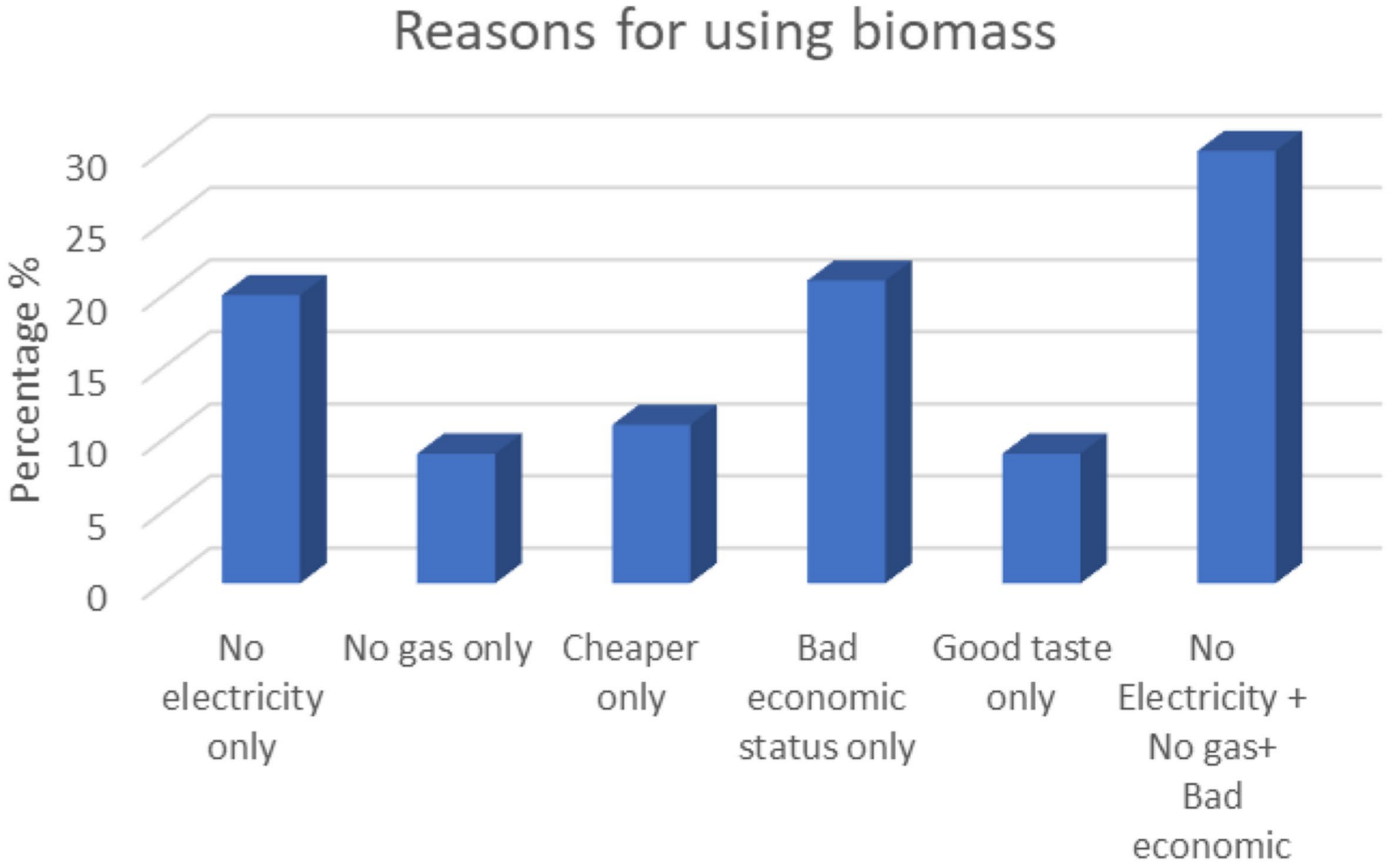
Reasons for biomass fuel use among study population.

Multiple factors, such as socioeconomic status, educational level, climatic conditions, and cooking habits, influence exposure to indoor air pollution from the combustion of solid fuels. Thus, the individual exposure–response relationship may be most directly influenced by the interaction of these factors with the source and the surrounding environment. The main reasons for using biomass fuel in households among the study population were due to precarious economic conditions (21%) followed by electricity shortage (20%), cheaper prices (11%), and shortage of gas (9%), as shown in Figure 2. Furthermore, 30% of respondents revealed that they used biomass due to a combination of reasons such as shortage of electricity and gas in addition to precarious economic conditions.

### Stove type and energy density

In the Gaza Strip, there are different types of stoves, such as a pit (a hole in the ground), a U-shaped construction made from mud, and three pieces of brick to provide the heat necessary for daily living requirements. The majority of households (48.2%) were found to use traditional stoves, cooking in the open with three stones fire, as shown in Table 3. A traditional clay oven without a chimney was used in 23.6% of the households and 23.6% used a traditional iron or clay oven with a chimney. The estimated weight of biomass used each time varied among the study population, with 16.4% of respondents using 1 kg of biomass daily, 32.7% of them using 2 kg, 26.4% using 3 kg, and 24.5% using more than 4 kg.

**Table 3:** Type of ovens and amount of biomass used by respondents.

Items	Frequency (%)
**Type of oven used for biomass**	
Open with three stones fire	53 (48.2)
Clay oven without chimney	26 (23.6)
Iron or clay oven with chimney	26 (23.6)
Both open oven and clay oven	5 (4.6)
**Estimated weight of biomass used each time**	
1 kg	18 (16.4)
2 kg	36 (32.7)
3 kg	29 (26.4)
4≥ kg	27 (24.5)
**Distance traveled from the house for collecting biomass**	
≤1 km	77 (70.0)
2 km	17 (15.5)
≥3 km	16 (14.5)

### The occurrence of common symptoms among respondents due to biomass use

As a result of strenuous work due to biomass collection and burning, people in general and women, in particular, can suffer from serious long-term physical damages, such as back pain. Approximately 46.4% of respondents had burned a part of their body due to using biomass, 58.2% had injured a part of their body due to cutting wood and 51.8% felt back pain due to carrying wood from long distances, as presented in Table 4. The reported back pain was probably related to the ergonomic position when carrying wood as well as due to the use of wood as household fuel; wood consumption faces a decreased burning efficiency, meaning that the respondents had to collect and carry more wood (24).

**Table 4:** Direct health issues from biomass collection and burning.

Items	Frequency (%)
**Burns in one part of the body**	
Yes	51 (46.4)
No	59 (53.6)
**Injury in one part of the body**	
Yes	64 (58.2)
No	46 (41.8)
**Had back pain**	
Yes	57 (51.8)
No	53 (48.2)

Burning biomass fuels leads to serious indoor air pollution (IAP) and gives rise to abundant amounts of poisonous emissions and inhalable particulates. The participants experienced different symptoms/morbidities such as dizziness, headache, eye irritation, nasal irritation, coughing, wheezing, bronchiolitis, and pneumonia. As seen in Table 5, the most common symptoms/morbidities were nasal irritation (71.8%), headache (66.4%), and dizziness (65.4%). Pneumonia was considered the least common with 17.3%, followed by wheezing with 41.8%.

**Table 5:** The occurrence of common symptoms/morbidities among respondents due to biomass use.

Symptoms/morbidity	Frequency (%)
**Dizziness**	
Present	71 (64.5)
Absent	39 (35.5)
**Headache**	
Present	73 (66.4)
Absent	37 (33.6)
**Eye irritation**	
Present	56 (50.9)
Absent	54 (49.1)
**Nasal irritation**	
Present	79 (71.8)
Absent	31 (28.2)
**Coughing**	
Present	52 (47.3)
Absent	58 (52.7)
**Wheezing**	
Present	46 (41.8)
Absent	64 (58.2)
**Bronchiolitis**	
Present	48 (43.6)
Absent	62 (56.4)
**Pneumonia**	
Present	19 (17.3)
Absent	91 (82.7)

### Association between the types, frequency, and location of biomass fuel used with common symptoms/morbidities among respondents

Tables 6, 7 show a comparison of symptoms/morbidities in different types, frequencies, and locations of biomass fuel use. The Chi-square test and Fisher’s exact test revealed a statistically significant difference in eye irritation and nasal irritation with the different types of biomass fuel (*p* = 0.01). A non-statistically significant difference between other symptoms/morbidities was found with the frequency of use and location where biomass was burned.

**Table 6:** Association between types, frequency, and location of biomass fuel used with common symptoms/morbid among respondents.

Variables	Dizziness	Headache	Eye Irritation	Nasal irritation	Coughing	Wheezing	Bronchiolitis	Pneumonia
	No. (%)	No. (%)	No. (%)	No. (%)	No. (%)	No. (%)	No. (%)	No. (%)
Total	71 (64.5)	73 (66.4)	56 (50.9)	79 (71.8)	52 (47.3)	46 (41.8)	48 (43.6)	19 (17.3)
**Type of biomass**								
Wood (34)	19 (55.9)	19 (55.9)	11 (32.4)	24 (70.6)	13 (38.2)	11 (32.4)	12 (35.3)	3.0 (8.8)
Coal (5)	3.0 (60.0)	2.0 (40.0)	2.0 (40.0)	2.0 (40.0)	2.0 (40.0)	1.0 (20.0)	0.0 (0.0)	0.0 (0.0)
Cardboard (6)	5.0 (83.3)	5.0 (83.3)	2.0 (33.3)	6.0 (100)	2.0 (33.3)	2.0 (33.3)	2.0 (33.3)	2.0 (33.3)
Mix of wood, cardboard, and dried grass (65)	44 (68.8)	47 (73.4)	41 (46.1)	47 (73.4)	35 (54.7)	32 (50.0)	34 (35.1)	14 (21.9)
*p*-value	0.36	0.09	0.01	0.01	0.39	0.30	0.06	0.271
**Frequency of use**								
Once a day (30)	15 (50.0)	16 (53.3)	14 (46.7)	20 (66.7)	15 (50.0)	13 (43.3)	14 (46.7)	7.0 (23.3)
More than once a day (38)	30 (78.9)	27 (71.1)	21 (55.3)	30 (78.9)	20 (52.6)	17 (44.7)	19 (50.0)	4.0 (10.5)
Once a week (20)	13 (65)	12 (60.0)	11 (55.0)	12 (60.0)	7.0 (35.0)	7.0 (35)	5.0 (25.0)	2.0 (10.0)
More than once a week (22)	13 (59.1)	18 (81.8)	10 (45.5)	17 (77.3)	10 (45.5)	9.0 (40.9)	10 (45.5)	6.0 (27.3)
*p*-value	0.09	0.15	0.85	0.39	0.63	0.92	0.31	0.242
**Location where biomass is burned**								
Inside the home (31)	19 (61.3)	24 (77.4)	15 (48.4)	21 (67.7)	14 (45.2)	16 (51.6)	15 (48.4)	9.0 (29)
Outside the home (79)	52 (65.8)	49 (62.0)	41 (51.9)	58 (73.4)	38 (48.1)	30 (38.0)	33 (41.4)	10 (12.7)
*p*-value	0.65	0.12	0.74	0.55	0.83	0.20	0.67	0.052

Data are expressed as percentages for different categorical variables. The Chi-square test and Fisher’s exact test were used.

**Table 7:** Logistic regression analysis results for experienced symptoms/morbidities and independent variables.

Variable	Eye irritation OR [95% CI]	*p*-value	Nasal irritation OR [95% CI]	*p*-value	Pneumonia OR [95% CI]	*p*-value
**Type of biomass**						
Mix of wood, cardboard, and dried grass	Ref		Ref		–	–
Wood	1.31 (1.54–8.99)	0.01	0.06 (0.42–2.66)	0.89	–	–
Coal	0.98 (0.41–17.18)	0.30	1.34 (0.59–24.8)	0.15	–	–
Cardboard	1.271 (0.60–0.20.98)	0.16	1.78 (0.91–5.23)	0.08	–	–
**Windows during biomass burning process**						
Close	Ref				Ref	
Open	0.14 (0.03–0.68)	0.01	–	–	5.53 (1.60–19.0)	0.01

The results of logistic regression showed that the participants who used wood fuel had a higher chance of having eye irritation than those who used a mix of wood, cardboard, and dried grass (OR = 1.316; 95% CI = 1.54–8.99).

### Association between duration, estimated weight used each time, and use of windows during the burning process with common symptoms/morbidities among respondents

Tables 7, 8 show a comparison of symptoms/morbidities related to the duration of biomass fuel use, estimated weight used each time, and window status (whether windows were opened or closed during the burning process). The Chi-square test and Fisher’s exact test revealed a statistically significant difference in eye irritation and pneumonia with windows status during the burning process (*p* < 0.05). Non-statistically significant differences between other symptoms/morbidities were found with duration and the estimated weight used each time.

**Table 8:** Association between duration, estimated weight used each time, and open windows during the burning process with common symptoms/morbidities among respondents.

Variables	Dizziness	Headache	Eye Irritation	Nasal irritation	Coughing	Wheezing	Bronchiolitis	Pneumonia
	No. (%)	No. (%)	No. (%)	No. (%)	No. (%)	No. (%)	No. (%)	No. (%)
Total	71 (64.5)	73 (66.4)	56 (50.9)	79 (71.8)	52 (47.3)	46 (41.8)	48 (43.6)	19 (17.3)
**Duration of biomass fuel use/day**								
1 h or less (26)	12 (46.2)	14 (53.8)	10 (38.5)	17 (65.4)	10 (38.5)	5.0 (19.2)	8.0 (30.8)	3.0 (11.5)
2 h (49)	34 (69.4)	38 (77.6)	26 (53.1)	36 (73.5)	26 (53.1)	27 (55.1)	25 (51.0)	11 (22.4)
3 h or more (35)	25 (71.4)	21 (60.0)	20 (57.1)	26 (74.3)	16 (45.7)	14 (40.0)	15 (42.9)	5.0 (14.3)
*p*-value	0.08	0.07	0.73	0.50	0.50	0.01	0.25	0.425
**Weight of biomass used each time**								
1 kg (18)	10 (55.6)	10 (55.6)	8.0 (44.4)	15 (83.3)	9.0 (50.0)	5.0 (27.8)	9.0 (50.0)	3.0 (16.7)
2 kg (36)	21 (58.3)	24 (66.7)	15 (41.7)	24 (66.7)	20 (55.6)	16 (44.4)	16 (44.4)	5.0 (13.9)
4≥ kg (56)	40 (71.4)	39 (69.6)	33 (58.9)	40 (71.4)	23 (41.1)	25 (44.6)	23 (41.1)	11 (19.6)
*p*-value	0.30	0.58	0.25	0.46	0.35	0.46	0.78	
**Windows during biomass burning process**								
Open (97)	66 (68.0)	66 (68.0)	54 (55.7)	70 (72.2)	47 (48.5)	38 (39.2)	42 (43.3)	13 (13.4)
Closed (13)	5.0 (38.5)	7.0 (53.8)	2.0 (15.4)	9.0 (69.2)	5.0 (38.5)	8.0 (61.5)	6.0 (46.2)	6.0 (46.2)
*p*-value	0.06	0.33	0.01	0.82	0.56	0.14	0.84	0.010

Data are expressed as percentages for different categorical variables. The Chi-square test and Fisher’s exact test were used.

The results of logistic regression showed that the participants who opened windows during the burning process of biomass fuel had a higher chance of having eye irritation (OR = 0.145; 95% CI = 0.030–0.688). In addition, participants who opened windows during biomass fuel burning were five times more likely of developing pneumonia than those who closed windows (OR = 5.53; 95%CI = 11.60–19.0).

## Discussion

This community survey illustrates the impact of household cooking fuel types in the Gaza Strip. The Gaza Strip has the sixth highest population density in the world. Moreover, the living situation inside refugee camps is characterized by high population density and poor conditions (25). Rogge (26) reported that the Palestinian refugee situation is the most protracted of all the refugee crises. As shown in Table 1, most respondents had a total monthly family income of 400$ US dollars (69.1%). Poverty, unemployment, and the unskilled labor status of those living in the camps are assumed to have an impact on their health.

Several studies revealed that the choice of fuel depends on the availability and access to biomass and modern energy (27–29). As shown in Table 2, household cooking fuels are often collected from the local environment due to the availability of agricultural lands as well as the participants’ economic status. Households in the Al-Maghazi area generally suffer from poverty, which is inextricably linked to the use of biomass. Moreover, the availability of biomass taken from the local environment may be linked to its heavy usage. According to the WHO, the price of using biomass energy is simply the labor required in collecting it (30). Often, households preferred energy-efficient fuels, such as liquefied petroleum gas (LPG), and electricity. However, Gaza’s sole power plant (GPP) was forced to shut down completely after exhausting its fuel reserves and being unable to replenish them due to a shortage of funds in the last 15 years (31). Thus, electricity shortage, with limited electricity for up to 4 h per day, has increased the uncertainty for local households. The current situation has serious implications for health, sanitation, and the region’s water. Thus, the overall humanitarian situation will be affected.

Furthermore, cultural habits play an important factor and influence cooking practices, which in turn may affect the duration of cooking or the quantity of fuel used. In Palestine, most people cook three meals a day, which was the case for the present study’s respondents. Many families use a wooden stove for preparing traditional breads and hot drinks, such as coffee, due to their taste preferences (9%), as shown in Figure 2. This is considered as a main obstacle to discontinuing cooking by using fuelwood. These were the two most common reasons quoted by our study population for continuing its use, which is similar to the findings reported in a study conducted in Ethiopia and Kenya (32, 33).

In developing countries reliant on biomass, women and children are generally responsible for fuel collection. The average distance for biomass collection was 1 km per day for 70% of respondents, followed by greater than two kilometers or more per day for 30% of them, as presented in Table 3. Biomass collection time has a significant cost, which limits the opportunity for women to engage in income-generating activities, thus improving their economic conditions. In the Palestinian community, women are traditionally responsible for cooking and other household chores. Thus, exposure levels to indoor pollutants are usually much higher among women and young children. A child exposed to indoor air pollution is two to three times more likely to develop pneumonia, which is one of the diseases most responsible for young children’s death globally (34).

Moreover, one factor that increases the exposure level is the biomass-burning location. Several studies reported a higher proportion of families who used outdoor kitchens compared to our study (35, 36). Table 2 shows that 71.8% of respondents burned solid fuel outside their buildings, however, 88.2% of them left building windows open during the burning process. People spent most of their time inside buildings, thus their exposure to indoor pollutants effectiveness was high. Several studies revealed that high indoor/outdoor (I/O) ratios for several pollutants are more than 1, suggesting that the building facade may not prevent the infiltration of pollutants indoors (37–39).

The results of this study suggest that different symptoms, such as dizziness, headache, eye irritation, coughing, wheezing, bronchiolitis, and pneumonia, commonly occur among people exposed to cooking smoke. The associated symptoms were classified into two groups: respiratory symptoms (coughing, wheezing, rapid heart rate, and inflammation of the airways) and neurologic symptoms (headache, dizziness, difficulty remembering something or difficulty concentrating, nervousness, and stress). This result is further evidence that those residents performing cooking services (and thus being exposed to IAP for a longer time) suffer higher hazards caused by biomass use. Moreover, the result of this study is similar to the observations made by several studies conducted globally (40–42).

The results of logistic regression showed that the participants who used wood fuel had a higher chance of than those who used a combination of wood, cardboard, and dried grass (OR = 1.316; 95% CI = 1.54–8.99). Several pollutants are released into breathing zones from incomplete combustion of biomass, such as CO, NO_2_, particulate matter (PM), many precursor components of photochemical smog, and ozone (43). Moreover, the results of logistic regression showed that the participants who opened windows during the burning process of biomass fuel had a higher chance of developing eye irritation (OR = 0.145; 95% CI = 0.030–0.688). In addition, participants who opened windows during biomass fuel burning had five times more likely to develop pneumonia than those who closed windows (OR = 5.53; 95%CI = 11.60–19.0). Opening of windows increases symptoms since higher exposures are associated with increased ventilation and opening of windows. Pollutants can migrate from outdoors to indoors and indoor air sources can exacerbate indoor air pollution. Indeed, several studies revealed that indoor air pollution concentrations can exceed outdoor air pollution concentrations (44–46). Furthermore, the burning of biomass fuel outside the surveyed buildings in this study was done at a very close distance to the building façade instead of building envelope, which influenced the indoor pollutant concentrations. Women, children, and elderly refugees have particularly high exposure to household biomass pollutant emissions because of their higher inhalation of household smoke, more vulnerable airways, and the fact that, in this region, they culturally spend more time at home. There is evidence that the lung function growth of children in high-polluted areas is significantly lower than those who live in less-polluted regions (47). The typical 24-h level of PM_10_ in homes that make use of biomass fuels in developing countries ranges from 300 to 3,000 (μg/m^3^), peaking at 10,000 μg/m^3^ while cooking is ongoing (48–50). Incomplete burning of biomass produces highly toxic and health-damaging pollutant particles, such as polycyclic aromatic hydrocarbons (PAHs), that penetrate the alveolar region and enter the human bloodstream. A meta-analysis of 24 studies found that exposure to PAHs emitted from biomass had a higher chance of developing pneumonia than those not exposed to PAHs (51).

### Limitations

The level of use of biomass may have been influenced by the season in which the survey was conducted, and a single measurement may lead to misclassification even among lifetime biomass users. Furthermore, the research was unable to capture data on indoor concentrations, ventilation, and individual exposure levels to smoke. Additionally, the concentrations of pollutants outside were not determined for this research, which would have provided insight into how outdoor air impacts indoor air quality. However, because there was no industrial pollution in the study region, this was lessened. This study is also subject to the limitations of survey questionnaires, including recall bias and exposure measurements that were limited to a single point in time.

The lack of information on accessibility to healthcare, which may be linked to the severity of health symptoms, is another limitation of this research. It is possible that some of the respondents who reported having health issues were not actually cooking with biomass fuel or had never been exposed to biomass smoke. Finally, we think that some households in the Gaza Strip urban area combine the use of biomass fuels for cooking and energy requirements with the use of other cleaner fuels. To reduce the possibility of residual confounding factors and increase the internal validity of our findings, we restricted the recruitment of our participants to a neighborhood that is largely homogeneous in terms of socioeconomic status, house construction, and access to healthcare. Further research is, therefore, necessary to determine whether the patterns of risk for health symptoms seen in this study are comparable to patterns in urban areas with diverse household characteristics or whether the risk may be mediated by several other variables.

## Conclusion

The study findings provide baseline information regarding the prevalence of biomass fuel use for cooking at the household level in the Palestinian Al-Maghazi refugee camp, as well as the association of biomass fuel use with sociodemographic characteristics, and self-reported health conditions in the Gaza strip, Palestine. The results have shown that the majority of households use wood and dried leaves as cooking fuel due to precarious economic conditions and electricity shortages. The results of this study suggest that a spate of different symptoms, such as dizziness, headache, eye irritation, coughing, wheezing, bronchiolitis, and pneumonia, commonly occur among people exposed to cooking smoke. The findings from this study suggest that there is an urgent need for public information campaigns designed to inform people about the risks of exposure to household harmful air pollutants and motivate more community development programs for poverty reduction.

## Data availability statement

The original contributions presented in the study are included in the article/supplementary material, further inquiries can be directed to the corresponding author.

## Ethics statement

The studies involving human participants were reviewed and approved by the study was approved by the ethics committee at Al-Israa University. In addition, informed consent was collected from all participants before completing the questionnaire. The patients/participants provided their written informed consent to participate in this study.

## Author contributions

ME and AA contributed equally to the study and the final manuscript reading and approval. All authors contributed to the article and approved the submitted version.

## Conflict of interest

The authors declare that the research was conducted in the absence of any commercial or financial relationships that could be construed as a potential conflict of interest.

## Publisher’s note

All claims expressed in this article are solely those of the authors and do not necessarily represent those of their affiliated organizations, or those of the publisher, the editors and the reviewers. Any product that may be evaluated in this article, or claim that may be made by its manufacturer, is not guaranteed or endorsed by the publisher.
